# Effects of Lipoic Acid on Ischemia-Reperfusion Injury

**DOI:** 10.1155/2021/5093216

**Published:** 2021-10-05

**Authors:** Yueming Ding, Yiming Zhang, Wunong Zhang, Jia Shang, Zhenxing Xie, Chaoran Chen

**Affiliations:** ^1^Institute of Nursing and Health, School of Nursing and Health, Henan University, Kaifeng, Henan, China; ^2^College of Educational Sciences, Henan University, China; ^3^School of Kaifeng Culture and Tourism, Henan, Kaifeng, China; ^4^School of Basic Medicine, Henan University, Henan, Kaifeng, Jinming Avenue, 475004, China

## Abstract

Ischemia-reperfusion (I/R) injury often occurred in some pathologies and surgeries. I/R injury not only harmed to physiological functions of corresponding organ and tissue but also induced multiple tissue or organ dysfunctions (even these in distant locations). Although the reperfusion of blood attenuated I/R injury to a certain degree, the risk of secondary damages was difficult to be controlled and it even caused failures of these tissues and organs. Lipoic acid (LA), as an endogenous active substance and a functional agent in food, owns better safety and effects in our body (e.g., enhancing antioxidant activity, improving cognition and dementia, controlling weight, and preventing multiple sclerosis, diabetes complication, and cancer). The literature searching was conducted in PubMed, Embase, Cochrane Library, Web of Science, and SCOPUS from inception to 20 May 2021. It had showed that endogenous LA was exhausted in the process of I/R, which further aggravated I/R injury. Thus, supplements with LA timely (especially pretreatments) may be the prospective way to prevent I/R injury. Recently, studies had demonstrated that LA supplements significantly attenuated I/R injuries of many organs, though clinic investigations were short at present. Hence, it was urgent to summarize these progresses about the effects of LA on different I/R organs as well as the potential mechanisms, which would enlighten further investigations and prepare for clinic applications in the future.

## 1. Introduction

Ischemia refers to decreased blood perfusions or total occlusions in tissues or organs. It causes deficiencies of oxygen and other nutrients and massive accumulations of metabolic wastes. Ischemia may be caused by many reasons, such as occlusion (e.g., thrombi), trauma, organ transplantation, and atherosclerosis. Ischemia can quickly result in tissue necrosis if there are no timely perfusions [1]. Early reperfusion is the preferred intervention to prevent the pathological process. However, the reperfusion after ischemia often caused inflammations and oxidative damages via oxidative stress due to increased levels of reactive oxygen/nitrogen species [2]. Enzymes (e.g., sodium-hydrogen exchanger pumps), molecules (e.g., DNA), cell damages (even deaths), and tissue dysfunctions ensued [3]. As a result, I/R injury caused further damages of many tissues (e.g., liver, heart, lung, and kidney). Still, the detailed mechanism of I/R injury was not fully clarified despite the above potential reasons. The short treatment window period affected the effects of reperfusion therapy [4], and developing new drugs became especially important [5]. Therefore, it was urgent to develop new agents with better effects and lower side effects to prevent I/R injury. Recently, many foodborne active substances had been developed for the prevention of I/R injury (e.g., coenzyme Q10 [6], resveratrol [7], and n-3 polyunsaturated fatty acids [8]). Due to these substances (especially those also existing in the body) owning the property of low security risks, it provided a prospective research area for developing more substances to prevent I/R injury.

## 2. Brief of Lipoic Acid

### 2.1. The Source, Biosynthesis, and Metabolism of LA

LA (also called as alpha-lipoic acid, thioctic acid, 6,8-dithiooctanoic acid, etc.), as an endogenous active molecule, was firstly isolated in 1951 by Reed et al. [9]. Afterwards, LA was confirmed as a vital cofactor of several enzymes (the pyruvate dehydrogenase complex, the *α*-ketoglutarate dehydrogenase complex, the branched-chain *α*-ketoacid dehydrogenase complex, the 2-oxoadipate dehydrogenase complex, and the glycine cleavage system) necessary for aerobic metabolism in mitochondria and the synthesis of nucleic acids [10]. Its natural molecule of (R)-(+)-enantiomer is mostly present in the form of combining with lysine residues of proteins in a wide variety of foods of plants (e.g., spinach and broccoli) and animals (e.g., kidney, heart, and liver) [11]. Free LA is mainly from chemical synthesis, which produced two enantiomers (R)-(+)-LA and (S)-(-)-LA. The former is the preferred form for the purpose of nutrition and therapy [12]. Na^+^-dependent multivitamin transporter is responsible for LA uptake in the gastrointestinal track and its transportation into tissues from blood [13]. Then, LA plays its biological roles.

Endogenous LA is made from octanoic acid in mitochondria of eukaryotes. The octanoyl moiety of octanoyl-acyl carrier protein formed in the process of fatty acid synthesis is first transferred to the lysine of the H protein of the glycine cleavage system by octanoyltransferase, and then, two hydrogens (octanoyl positions 6 and 8) are replaced with sulfur groups, forming LA which can be reduced to dihydrolipoic acid (DHLA, the more bioactive form of LA) by the LA synthetase. Then, dihydrolipoyl moiety is transferred to lysine residues on the E2 components of the *α*-ketoacid dehydrogenase multienzyme complexes by the dihydrolipoamide dehydrogenase [10]. For free LA, the major metabolic fate is *β*-oxidation [14]. Combined form of LA *in vivo* can be metabolized as diversified forms: the conjugation of unmodified LA to glycine, tetranorlipoic acid, bisnorlipoate, and *β*-hydroxy-bisnorlipoate and the sulfoxide and S-methylation of the sulfide from its sulfur atoms in mammals [10, 14].

### 2.2. Biological Functions of LA

**Figure 1:**
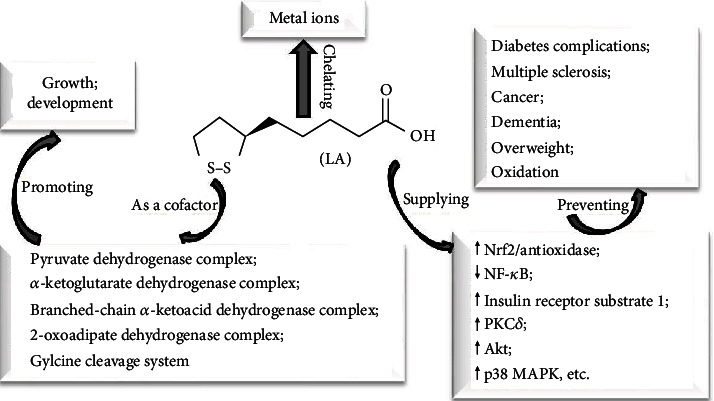
The biological functions of LA and potential mechanisms. Akt: protein kinase B; LA: lipoic acid; p38 MAPK: mitogen-activated protein kinase p38; Nrf2: nuclear factor erythroid 2-related factor 2; PKC: protein kinase *C.*

In physiological condition, endogenous LA can meet the need of fundamental functions mentioned above, with rare deficiency cases in human [15]. Moreover, many studies had shown that dietary supplements (in the free form of LA from chemical synthesis) can provide more additional effects, for example, owning antioxidant activities (through restoring reduced glutathione/glutathione [16]; disulfide regenerating vitamin C and vitamin E [17]; chelating iron, copper, manganese, and zinc [18]; inhibiting nuclear factor-*κ*B (NF-*κ*B) [19]; and activating nuclear factor erythroid 2-related factor 2(Nrf-2)/antioxidase [20]), improving cognition and dementia [21], controlling weight [22], and the treatment and prevention of other diseases (e.g., multiple sclerosis [23], diabetes complication [24], and cancer [25]) (Figure 1). There are more signaling pathways that LA acted on (e.g., insulin receptor substrate 1 [26], protein kinase C *δ* (PKC*δ*) [26], extracellular-regulated kinase 1/2 (Erk1/2) [27], phosphatidylinositol 3-kinase (PI3K)/protein kinase B (Akt), and mitogen-activated protein kinase p38 (p38 MAPK) [28]. Meanwhile, LA can exert effects on almost every organ in the body (e.g., heart, kidney, and intestine) [29]. The elevated plasma LA levels after LA supplements may be responsible for these effects [30]. However, it seems impossible for exogenous LA to utterly replace the endogenous synthesis because it cannot be incorporated into LA-dependent enzymes, which is necessary for normal growth and development [31]. Still, LA had been approved for treating diabetic neuropathies by prescription in Germany [17]. Even, it is also available as an over-the-counter nutritional supplement in the United States. It indicated that free LA may own biological functions almost utterly consistent with the combined form of LA. Studies showed that it was safe for LA at the dosage of 600 mg/kg via a single intravenous administration without serious side effects [32, 33], exception of fewer exceptions in pregnancy [31] and lactation [34], together with certain risks of high doses in children [35] and the aging animal model [36]. And, there was a report showing that oral administration of 1,800 mg for 6 months had not any significant adverse side effects [37]. Therefore, it was attracting more researchers to explore potential biological functions of LA.

Many studies using LA to prevent I/R injury had been carried out in recent ten years though scientists had noticed these effects twenty-five years ago [38]. Increasing studies showed that supplements with free LA can play the vital role in attenuating I/R injuries of different organs and tissues. Meanwhile, more mechanisms apart from LA antioxidation involved in these processes were updated rapidly. Therefore, it was necessary to timely review these data in order to provide insights for further mechanism explorations and clinic investigations.

## 3. The Effects of LA on I/R Injuries of Different Organs

### 3.1. The Protective Role of LA in Cardiac I/R Injury

Myocardial ischemia is caused by reduced coronary blood flow accompanied with electric, functional, metabolic, and structural abnormities [39]. Restoring blood supply is the first choice of intervention, or lack of oxygen and energy supplies would cause myocardial atrophies, apoptosis, and necrosis [40]. Although the reperfusion reduced infarct size and necrosis, it also brought about additional injuries to the myocardium (myocardial I/R injury). The molecular mechanism of myocardial I/R injury mainly lay in a series of pathological processes: Ca^2+^ oscillations between cytosol and mitochondria, opening of the mitochondrial permeability transition pore, the production of reactive oxygen species, and the effects of transferring pH correction between adjacent myocytes due to acidosis [41]. In addition, prothrombogenic factors, endothelial cell activations, and inflammatory responses also contributed to the final damage of myocytes (even death) [42]. The myocardial I/R process was also considered as the main pathological manifestation of coronary artery disease [43]. Therefore, exploring cardioprotective bioactive agents was needed for attenuating myocardial I/R injury. In fact, the role of LA in myocardial I/R injury had been showed in an early study. The study [44] found that myocardial I/R can induce the loss of native LA in *α*-ketoglutarate dehydrogenase and the decline in *α*-ketoglutarate dehydrogenase activity. It was considered as the result of lipid peroxides reacting with LA residues on the E2 subunit of *α*-ketoglutarate dehydrogenase under free radicals produced in myocardial I/R process [45]. Therefore, supplements with LA may be a potential choice for attenuating myocardial I/R injury. Recently, studies demonstrated that LA supplements attenuated myocardial I/R injury *in vivo* and *in vitro* (Table 1). Main mechanisms included reducing oxidative stress via activating the PI3K/Akt/Nrf2 pathway and restoring alpha-tocopherol, inhibiting autophagy, restoring PKC*ε*-dependent aldehyde dehydrogenase 2 (ALDH2) activities, and activating potassium ATP-sensitive channels.

Although the beneficial effects of LA on attenuating myocardial I/R injury are partly attributed to increased levels of other antioxidative agents (e.g., vitamins E and C) [46], sometimes, it was still essential for combining LA and these agents in preventing myocardial I/R injury because the combination would overcome the potential defects of applying LA alone in specific conditions (e.g., increased lipid peroxide levels in the heart of an aging person [36]). A study [47] showed that cardiac performances of aging rats after I/R were notably improved and lipid peroxidation in the heart was remarkably decreased after the supplement with vitamin E and LA. In addition, there was an enhancing effect for DHLA on vitamin E attenuating myocardial I/R injury [48]. The study also found that this interaction was independent on their concentrations. However, whether there were other potential mechanisms still needed further investigations.

### 3.2. The Protective Role of LA on Renal I/R Injury

Renal I/R injury usually occurred under some medical conditions (e.g., shock, sepsis, hypoperfusion, and renal transplantation) [59, 60]. These conditions caused direct damages to tubules, blood vessels, and glomeruli. Meanwhile, oxidative stress, inflammation, and mitochondrial dysfunction together contributed to acute kidney injury, which led to the abrupt decline of kidney function and increased mortality [61]. It was very vital to improve blood supplies and reduce pathological damages in the process of renal I/R. However, there were unmet medicines by the present day. It was critical to develop safe and effective strategies before renal I/R lesions. Encouragingly, some studies had shown that LA pretreatments at shorter time before I/R can attenuate renal I/R injury whenever in both lateral and bilateral animal models (Table 2). Renal function, histological lesions, and molecular indexes (e.g., aquaporins, sodium transporters, creatinine, and urea) were restored to varying degrees. The mechanisms involved included the elevation of antioxidative abilities and the reduction of inflammation and necrosis through regulating some signaling pathways (e.g., maintaining the normal status of arginine vasopressin/cAMP, nitric oxide/cGMP, and endothelin (ET) systems; depressing ET-1; increasing matrix metalloproteinases-2 and -9 activities; and decreasing metalloproteinases-1 and -2 levels). Based on these studies, the pretreatment with LA for a long time before renal I/R may have better effect and may be considered in further studies. And clinic investigations should also be carried out for prospective applications in patients that suffered from renal I/R injury.

### 3.3. The Protective Role of LA on Hepatic I/R Injury

Liver ischemia is secondary to some pathologies (e.g., hemorrhagic shock and resuscitation [68]) and surgeries (e.g., liver resections [69] and liver transplantation [70]). Although, to some extent, the liver is tolerant of ischemia injury in the initial phase, the productions of xanthine oxidase and NADPH oxidase in liver cells drive more serious damages in the second phase of reperfusion [71]. The two-stage hepatic I/R injury was still the vital cause of liver surgical failure (e.g., hepatic resection and liver transplantation) and finally increased liver morbidity and mortality [72]. Reactive oxygen species and activation of inflammatory played the key role in the pathology of hepatic I/R injury [73]. It had previously demonstrated that LA can be used for curing liver-related diseases (e.g., hepatic cirrhosis, hepatomegaly in hepatic intoxication, alcohol clearance, and increased glucose disposal in the liver) [34]. Recent studies showed that LA can attenuate hepatic I/R injury in clinical and animal trials (Table 3). In two cases of human liver transplantation and resection, LA pretreatments (intravenous injections of 600 mg) remarkably reduced manifestations of postreperfusion syndrome via blocking oxidative stress and apoptosis. In animal models, LA treatments also alleviated hepatic I/R injuries in functions and pathological constructs. The mechanism included scavenging radicals, increasing antioxidant enzyme activities, inhibiting inflammatory reaction via activating the PI3-kinase/Akt pathway, inhibiting NF-*κ*B P65 and macrophage inflammatory protein-2 (MIP-2), and preventing hepatocyte deaths caused by apoptosis and necrosis. Therefore, LA was a prospective agent for preventing hepatic I/R injury in further clinical applications.

### 3.4. The Protective Role of LA on Cerebral I/R Injury

Cerebral ischemia, resulting from pathologies of brain blood vessels (occlusion or rupture) and cardiac dysfunctions [82], caused brain damages (e.g., neuronal deaths of adults [83] and hypoxic-ischemic encephalopathy of infants [84]). The morbidity and mortality of cerebral ischemia can be controlled to a certain degree by the timely reperfusion. However, cerebral reperfusion further aggregated cerebral injuries via inducing inflammation, oxidative stress, and the breakdown of the blood-brain barrier [85, 86]. Therefore, it is vital to explore more strategies to protect cerebral functions and pathological developments in cerebral I/R. Because LA can cross the blood-brain barrier easily without any serious side effects [87], it was considered an effective candidate to attenuate numerous neurodegenerative disorders via its antioxidative action [88]. It had previously been proven that LA pretreatment significantly protected cerebral focal and forebrain ischemia [89]. Further, studies *in vivo* and *in vitro* had shown that LA treatments restored neurological structures and functions in cerebral I/R via attenuating oxidative damage mediated by the Nrf2/heme oxygenase-1 (HO-1) pathway and reduced cerebral infarct volumes, apoptosis, and inflammation reaction via inhibiting the Toll-like receptor 4 (TLR4)/myeloid differentiation primary response 88 (MyD88)/NF-*κ*B pathway and Akt/mammalian target of rapamycin (mTOR) (Table 4).

LA also produced synergistic effects against cerebral I/R injuries when combined with other agents. For instance, the pretreatment with LA (20 mg/kg) and vitamin E (50 mg/kg) can reduce neurological deficits and reactive gliosis and promote neuronal remodelings in cerebral I/R rats [90]. Another study showed that the pretreatment of LA for 2 h in a lower dose (50 *μ*M) together with other thiol-stimulating agents (e.g., ambroxol and enalapril) can significantly increase neuronal survivals in oxygen glucose deprivation/reperfusion of ex vivo rat organotypic hippocampal slice cultures [91], despite no effects while it being used alone. Further, the cotreatment with LA and other agents reduced not only the effective dosage of LA but also that of other agents. Therefore, it would help reduce the potential toxicities of these agents when it was used at a noneffective low dose together with them. Two previous studies [92, 93] had shown that LA administrations at a very low dose (0.005 mg/kg) can significantly enhance the effects of two agents (apocynin and resveratrol) at very low doses (0.05 mg/kg and 0.002 mg/kg) prior to middle cerebral I/R. In contrast, the effective dosages of apocynin and resveratrol were 200- and 1000-fold decreases in the amount to achieve alike protective effects in cerebral I/R injuries while they were used alone. Therefore, the combination of LA with the two agents would significantly reduce occurrences of their potential side effects (e.g., possible prooxidant effects for apocynin and renal toxicity for resveratrol) at high dosages. Thus, to explore more combination of LA and other agents and corresponding mechanisms would be meaningful for clinic applications.

### 3.5. The Protective Role of LA on Spinal I/R Injury

Spinal I/R injury is usually induced by the cardiovascular surgery (e.g., the decompression of a previous compressed region of the spinal cord due to thoracoabdominal aortic interventions [109]). Spinal I/R injury further induces nerve injuries and damages of sensation and movement (even paraplegia) [110]. Oxidative stress, inflammation, and excitotoxicity were considered playing vital roles in spinal I/R injury [111]. In previous studies, some therapeutic measures had been used to cure spinal I/R injury (e.g., the preconditionings of hyperbaric oxygen [112] and remote ischemia [113], pharmacologic administrations such as progesterone [114], and the application of pluripotent stem cells [115]), but the effects of treatments were yet unsatisfied. Therefore, it was necessary to explore new treatments or agents for attenuating spinal I/R injury. LA, owning strong antioxidative abilities, had been used for curing spinal I/R injuries in animal models (Table 5). The studies showed that LA pretreatments can attenuate spinal neurological injuries via reducing oxidative damages and inflammation. The effect was related to the time point of exposing to LA in advance (e.g., LA pretreatments exhibiting poorer effects on spinal I/R injuries at 5 and 10 min before ischemia compared with these at longer time points from Table 5). However, further studies and clinical investigations were needed for potential applications of LA in attenuating spinal I/R injury.

### 3.6. The Protective Role of LA on Intestinal I/R Injury

Compared with other organs, the intestine is more sensitive to I/R injury [116]. Intestinal I/R injury occurred in many cases such as trauma, strangulated intestinal obstruction, acute mesenteric ischemia, thrombosis of the mesenteric artery, shock, incarcerated hernia, hemorrhagic, and surgical procedures (e.g., small intestinal transplantation, cardiopulmonary bypass, or abdominal aortic aneurysm) [117–120]. The high mortality [121] of intestinal I/R injury still occurred due to inflammation and oxidative stress though many intervention efforts had been made. It is essential to constantly search for more prevention strategies. Several studies showed that either early or simultaneous treatments of LA attenuated intestinal I/R injury. The difference between the two kinds of treatments was just that the simultaneous treatment of LA together with ischemia occurrence produced less protective effects on intestinal I/R injuries [122] compared to better protective effects exhibited by both LA preconditioning [123] and the subsequent continuous administration [124]. However, the defect of the former treatment can be offset through its combination with other agents (e.g., anti-inflammatory drug: ebselen). The mechanism of LA attenuating intestinal I/R injury in these studies can be attributed to its abilities of antagonizing oxidative damages and these anti-inflammatory effects of ebselen. Therefore, combined effects of LA and other drugs possibly provided an alternative treatment strategy for intestinal I/R injury.

### 3.7. The Protective Role of LA on Limb I/R Injury

Limb ischemia occurred in the lower extremities secondary to atherosclerotic vascular stenosis and other pathologies (e.g., aneurysms with intraluminal thrombus) [125]. Although there are lower incidences and longer reperfusion window periods [126], untimely reperfusion treatments yet induce irreversible tissue damages from skin numbness and paralysis of the musculature to limb loss and even death [127]. Treatment options had shifted from surgeries to endovascular therapies, which made preconditioning with active agents more vital for preventing limb I/R injury. Studies had shown that LA treatment can attenuate functional damages in the lower extremities of some pathologies (e.g., diabetic foot syndrome [128] and the polyneuropathy of patients with hemoblastosis [129]), indicating possible preventive and protective efforts of LA on limb I/R injury. A study [130] showed that LA treatment (24 mg/kg, i.p) after 4 hours of ischemia and at the onset of 24-hour reperfusion decreased the levels of muscle tumor necrosis factor-alpha and oxidative stress indexes in muscles of hind limb I/R model rats. Another study [55] showed that LA treatment (8.3 mM) along with 30 min reperfusion after 4 h hind limb ischemia (isolated rat hind limbs) also recovered the function (contractile and flexibility). The protective effect of LA on limb I/R injury was of inconsistent efficacies, depending on lasting time of the ischemia. For instance, intraperitoneal (i.p) pretreatments of LA (100 mg/kg daily for 3 days) improved distal sensory conductions of the sciatic-tibial nerves in the right hind limb artery I/R (3 h/7 d) model rats [131]. However, the ischemic nervous fiber degeneration was not attenuated by LA if ischemia time was prolonged to 5 h. It indicated that the period of ischemia may be the key factor, which aggravated I/R injury and made the injury hard to be reversed. However, whether long-term preconditionings with LA can attenuate these I/R injuries experiencing long-term ischemia needed further studies. LA treatment also attenuated the injuries of other tissues caused by limb I/R. A recent study [100] showed that limb I/R induced the degenerative injury of rat hippocampal CA3 neurons (e.g., the reduction of light neuron number, surface areas of neurons, and the thickness of the CA3 layer together with increased capillary lesions), and an injection of LA (100 mg/kg, i.p) for 7 d significantly ameliorated these damages. The result indicated the potential application of LA in both nervous system and peripheral tissues simultaneously induced by limb I/R.

### 3.8. The Protective Role of LA on I/R Injury of the Gonad

Gonadal ischemia is usually the emergency occurring in males (e.g., testis ischemia) or females (e.g., ovarian ischemia). Torsion is the main reason inducing ischemia in the process. For instance, testis ischemia is frequently caused by the rotation of spermatic cord and the involved patients mostly come from adolescents under 30 years old [132]. Ovarian ischemia is also caused by the torsion, which results from ovarian diseases, surgery, traumas, or pregnancy [133]. Due to acute pains in gonadal ischemia, the two kinds of I/R of gonad needed emergency treatments of surgical detorsion to restore blood perfusion to the gonad. If the reperfusion was not carried out timely, further dysfunctions of the gonad occurred, for example, apoptosis, testicular atrophy, and disturbances of spermatogenesis in males [134] and ovary edema, bleeding, and necrosis in females [135]. Even the successful surgical intervention cannot utterly restore the gonadal dysfunctions resulting from oxidative stress and inflammatory response after reperfusions [136, 137]. Therefore, it must be considered to develop other interventions of drugs and bioactive agents. Recently, several studies had been reported about protective efforts of LA treatments on I/R injury of the gonad. First, LA treatment can attenuate testicular I/R injury in males. The pretreatment with LA injections (12 mg/kg, i.p at 21, 9, and 1 h before ischemia) reduced testicular I/R injury through decreasing lipid peroxidation and increasing total levels of antioxidant power in rat blood plasma and testis [138]. Another research [139] found that LA administration (100 mg/kg, i.p) at 30 min before ischemia also produced beneficial effects in protecting testicular I/R injury, which decreased apoptosis and oxidative damage, and increased antioxidative abilities were also responsible for the effects.

Meanwhile, LA treatment also reduced ovarian I/R injury in females. Cosar et al. [140] found that injections with LA (36 mg/kg/d, i.p) in 21, 9, and 1 hour before ischemia can prevent rat ovarian I/R injuries (3 h/3 h) through reducing oxidative damage (downregulated malondialdehyde, xanthine oxidase, and nitric oxide; upregulated superoxide dismutase). By contrast, high dosage of LA pretreatment (100 mg/kg) in adnexa I/R injury ameliorated ovary dysfunctions (e.g., restored numbers of primordial follicles and primary follicles) based on attenuating total oxidant status and oxidative stress index [141]. However, bilateral ovarian damages may be differently affected by LA treatment while a lateral ovarian I/R occurred. LA treatment only attenuated the lateral ovarian damage undergoing I/R. The contralateral ovarian injury induced by increased sympathetic activity secondary to the I/R procedure was not restored [142]. Therefore, synergistic therapy in applying LA to prevent gonadal I/R injury may be considered to reduce the contralateral ovarian injury second to gonadal I/R.

### 3.9. The Protective Role of LA on Retinal I/R Injury

Retinal ischemia often occurred in some pathologies (e.g., glaucoma, diabetic retinopathy, ophthalmic artery, and some central retinal vein occlusion) and further developed into visual impairment and blindness [143]. Restored blood supplement after ischemia further induced injuries of retinal cells via producing reactive oxygen species. Inflammatory reactions and the damage [144] of retinal neurons [145] were considered as the key factors in structural and functional dysfunctions of the retina. Previous studies had shown that LA can play a protective role on retina pathologies. LA administration had been confirmed to protect the complications of retinal lesions in some diseases (e.g., glaucoma [146] and diabetes [147]). LA may be applied to attenuate retinal I/R injury. A study [148] showed that the injection with LA treatment (100 mg/kg∗day, i.p. for 11 days) rectified rat retinal I/R injuries (e.g., reducing amplitudes of the electroretinogram a and b waves, Thy-1(CD90) immunoreactivities, and retinal ganglion cell-specific mRNA levels and increasing bFGF and CNTF mRNA levels) via reducing nitric oxide synthase. *In vitro*, LA supplement (100 mM) in the cultural medium ameliorated the loss of GABA neurons in isolated rat retinas undergoinganoxic/reoxygenation. However, the mechanism was unclarified. Even in a long-term lead exposure model, the administration of LA (70 mg/kg, i.p) 24 hour prior to retinal I/R (2 h/5d) produced the anti-inflammatory effects via elevating levels of glial-derived neurotrophic factor (GDNF) and ciliary neurotrophic factor (CNTF) and reducing levels of glial fibrillary acidic protein (GFAP) [149]. Therefore, LA was a potential agent for preventing retinal I/R injury in future.

## 4. The Dose of LA in Attenuating I/R Injury and Potential Side Effects

The dosage of LA based on present data is of importance. The effects of LA on I/R injuries of different organs were mostly obtained from animal models. At present, the side effects were mainly affected by the dosage and the period of treatments. Firstly, for animal tests, the studies were mainly conducted in a short time prior to ischemia, in which even high doses of LA (up to 2000 mg/kg∗d) treatments nearly caused no acute toxicity except for some subhealthy signs in behavior (sedation, piloerection, hunched posture, and eye closure) [150]. However, a long-term treatment (more than 1 month) with middle doses of LA (e.g., 61.9 mg mg/kg∗d in females) caused single-cell necrosis of hepatocytes. Compared with animals, the dosages for human were relatively lower.For humans, a safe dose of LA as the health ingredient used in food supplements was recommended as a daily intake of 150-200 mg/d in Denmark [151], through the intakes of 300 to 1800 mg/d to improve symptoms of corresponding diseases had been applied. However, allergic reactions in the skin (e.g., hives and itchiness) and gastrointestinal symptoms (e.g., stomach ache, nausea, and diarrhea) often occurred in long-term applications [152–154]. Although there were no reports about the side effects of LA attenuating I/R injury at present and the dosages in present studies were relatively low, it should be considered the potential side effects of LA applying to prevent I/R injury. It was because of increasing risks of LA side effects in certain conditions (as discussed above in pregnancy [31], lactation [34], children [35], and the aging animal model [36]).Therefore, dosages of LA should be carefully explored for health maintenance and interventional therapies combined with different lengths of treatment time and the possible side effects.

## 5. Conclusion

**Figure 2:**
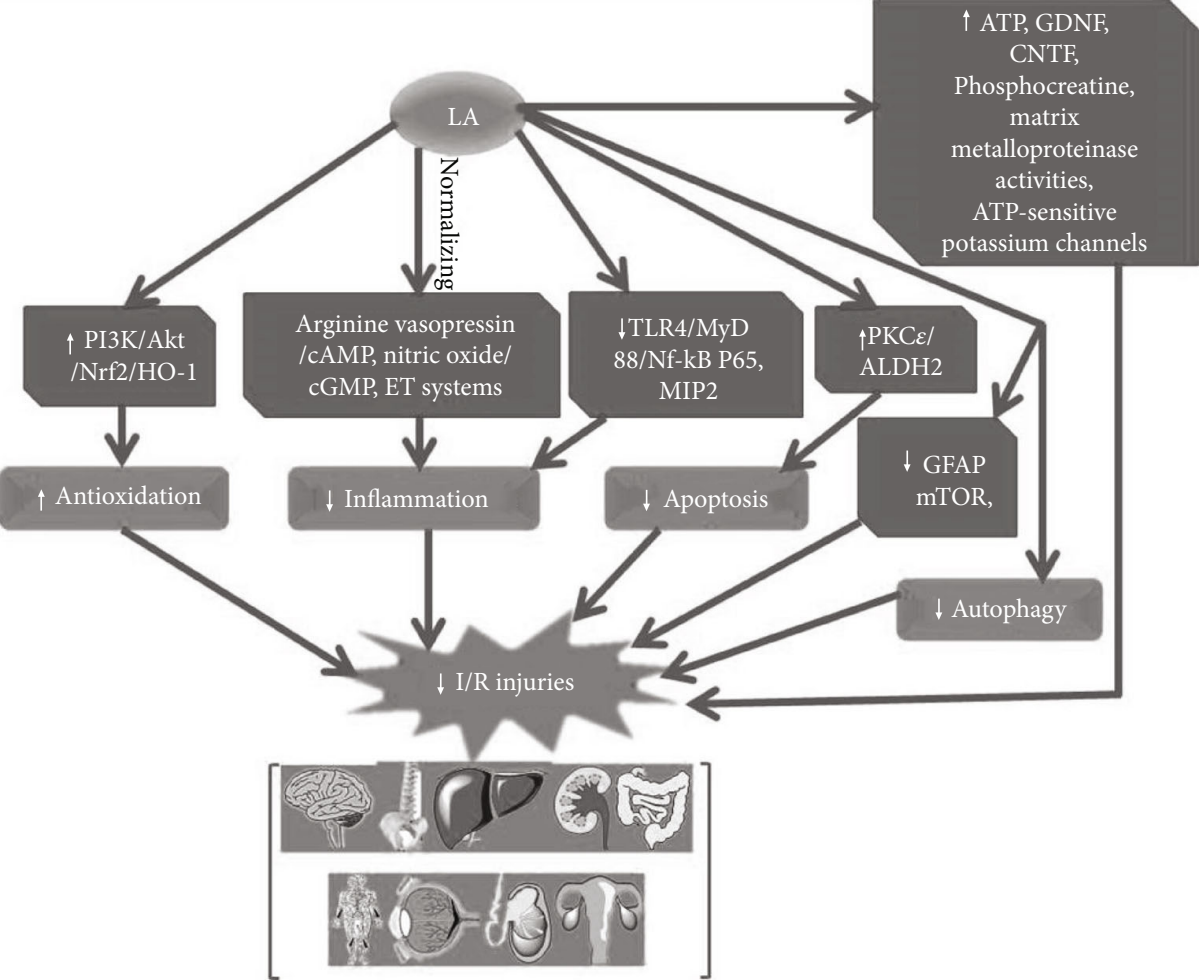
The mechanism of LA attenuating I/R injuries in the brain, spine, heart, liver, kidney, intestine, gonad, retina, and limb. Akt: protein kinase B; ALDH2: aldehyde dehydrogenase 2; CNTF: ciliary neurotrophic factor; ET: endothelin; GDNF: glial-derived neurotrophic factor; GFAP: reducing levels of glial fibrillary acidic protein; HO-1: heme oxygenase-1; LA: lipoic acid; MIP-2: macrophage inflammatory protein-2; mTOR: mammalian target of rapamycin; MyD88: myeloid differentiation primary response 88; Nrf2: nuclear factor erythroid 2-related factor 2; PI3K: phosphatidylinositol 3-kinase; PKC: protein kinase C; TLR4: Toll-like receptor 4.

Ischemia is caused by shortness of blood supplements, and the direct result is derangements of metabolic function (e.g., the decrease of ATP production) and dysfunctions of some gene expressions, such as the genes related to inflammatory response and genes producing free radicals due to hypoxia in mitochondria. Although reperfusion is always considered able to reduce further damages, I/R injury is unavoidable. Researchers constantly developed new interventions to overcome potential damages caused by reperfusion alone. LA, as a functional agent of food sources and an endogenous active substance, also owned additional biological functions and the roles for some diseases (improving cognition and dementia, controlling weight, and preventing multiple sclerosis, diabetes complications, and cancers) while supplying as a health component or the prescription. In the I/R process, the exhaustion of LA indicated its potential protective roles. Recently, studies had shown that supplements with LA can significantly attenuate I/R injuries in multiple organs (nervous system, heart, kidney, liver, intestine, gonad gland, retina, and limb). Apart from its antioxidative actions, other mechanisms also involved in protecting I/R injury (Figure 2) include the activation of potassium ATP-sensitive channels; the restoration of PKC*ε*-dependent ALDH2 activity/apoptosis inhibiting; autophagy inhibition; the activation of PI3K/Akt/Nrf2 nuclear translocation/HO-1; increases of ATP synthesis and phosphocreatine contents; the reduction of inflammatory reaction via maintaining the normal status of arginine vasopressin/cAMP, nitric oxide/cGMP, and ET systems; increasing matrix metalloproteinases-2, and -9 activities and decreasing metalloproteinase-1 and -2 levels; inhibiting TLR4/MyD88/NF-*κ*B P65 and MIP-2 mRNA; downregulating proapoptotic genes; reducing phosphate mTOR via Akt; elevating levels of GDNF and CNTF; and lowering levels of GFAP. Therefore, LA may be a prospective agent for preventing I/R injuries of multiple organs. Interestingly, the majority of these data was based on studies of pretreatments with LA. Therefore, fortifying the contents of lipoic acid in foods by chemical synthesis or supplying it from foods before I/R may provide a potential way to attenuate I/R injuries compared with the difficulty to prevent I/R injuries in treatments after I/R. In this respect, clinical investigations including the relationship between LA intake and the risk of I/R injuries as well as the data about LA intervention strategy as the health agent in human were very limited and may become a further research topic.

Besides, animal tests and human applications indicated potential side effects of LA. Thus, it was also an important concern about how to balance its protective actions and the potential side effects in I/R injury (with no reports). In conclusion, LA, as an endogenous and foodborne active agent, can attenuate I/R injuries of many organs, which widened the range of it as functional nutritional factors. The reasonable choices of administration time, dosages, and duration together with potential side effects should be considered in future investigations.

## Figures and Tables

**Table 1:** The effects of LA on myocardial I/R injury.

Species	I/R models	Treatments	Effects and mechanisms	Ref
Wistar rats	Left coronary artery I/R (15 min/30 min)	LA (10^−7^ M) from 15 min before ischemia to the end of reperfusion	Protecting cardiac I/R arrhythmias via its antioxidation and the activation of potassium ATP-sensitive channels	[49]
Sprague-Dawley rats	Isolated cardiac I/R (60 min/30 min)	LA (1, 5, 10∗10^−8^ M) for 10 min before ischemia	Reducing cardiac dysfunction and apoptosis via restoring PKC*ε*/ALDH2 activity	[50]
Sprague-Dawley rats	Isolated cardiac I/R (30 min/30 min)	LA (0.5 *μ*M) at 15 min before ischemia and during reperfusion	Improving cardiac hemodynamic function and damage via inhibiting mitochondrial O^·2-^ formation (by DHLA)	[51]
Wistar rats	Isolated cardiac I/R (30 min/30 min)	LA (50 mg/kg∗day, i.p for 7 d) before surgery	Increasing coronary flow via scavenging free radicals	[52]
Rat H9c2 cardiomyocytes	Hypoxia/reoxygenation (4 h/3 h)	LA (300 *μ*M) at 12 h before H/R	Protecting H/R injuries of cardiomyocytes by inhibiting autophagy	[53]
Sprague-Dawley rats	Left anterior descending coronary artery I/R (30 min/3 h)	LA (5, 10, 15, 25, or 50 mg/kg) via tail vein injection before I/R	Attenuating cardiomyocyte necrosis, apoptosis, and inflammation via activating PI3K/Akt/Nrf2 nuclear translocation/HO-1	[54]
Wistar rats	Hypoxia/reoxygenation (45 min/60 min) in isolated hearts	LA (0.3 mM) in the hypoxic period, at 15 min before reoxygenation	Accelerating the recovery of the aortic flow via increasing ATP synthesis and phosphocreatine contents	[55, 56]
Wistar diabetic rats	Global no-flow I/R (30 min/30 min) in isolated hearts	LA (100 mg/kg i.p., 5 times/w, for 8 w) before perfusion	Reducing serious reperfusion dysrhythmias	[57]
Sprague-Dawley rats	Aortic cannula I/R (40 min/20 min)	LA (1.65 g/kg∗d) pretreatment for 6 weeks	Decreasing lipid peroxidation products and the loss of alpha-tocopherol	[58]

**Table 2:** The effects of LA on renal I/R injury.

Species	I/R models	Treatments	Effects and mechanisms	Ref
Albino rats	Both renal I/R (1 h/2 h)	LA (100 mg/kg, i.p) at 2 days before I/R	Attenuating histopathological injury via reducing oxidative damage	[62]
Wistar albino rats	Right nephrectomy and left renal I/R (45 min/24 h)	LA (100 mg/kg, i.p) or saline twice, at 30 min before ischemia	Inhibiting neutrophil infiltration and inflammation generation, balancing the oxidant-antioxidant status	[63]
Sprague-Dawley rats	Bilateral renal I/R (45 min/24 h)	Pretreatment with LA (50 mg/kg i.p) for 2 weeks	Decreasing levels of creatinine and urea together with oxidative stress and inflammation; elevating GSH levels and activities of antioxidant enzymes	[64]
Albino Wistar rats	Right nephrectomy and the left renal I/R (1 h/24 h)	LA (100 mg/kg, i.p) 30 min before I/R	Reducing degradation of extracellular matrix and oxidative stress via increasing matrix metalloproteinases-2 and -9 activities and decreasing metalloproteinases-1 and -2 levels	[65]
Sprague-Dawley rats	Bilateral renal I/R (40 min/2 d)	LA (80 mg/kg i.p) at 48 and 24 h before ischemia and at 6 and 24 h after reperfusion	Protecting renal function (e.g., aquaporins and sodium transporters) via normalizing activities of local arginine vasopressin/cAMP, nitric oxide/cGMP, and ET systems	[66]
Sprague-Dawley rats	Right nephrectomy and left renal I/R (45 min/24 h)	LA (10 or 100 mg/kg, i.p) at 24 and 1 h before ischemia	Attenuating the deterioration of renal function; repressing tubular necrosis, proteinaceous casts in tubuli, and medullary congestion through suppressing ET-1	[67]

**Table 3:** The effects of LA on hepatic I/R injury.

Subjects	I/R	Treatments	Effects and mechanisms	Ref
Human	Hepatic I/R (2 h/2 h); blood samples (before; 1, 2, 3, 7, and 30 d after liver transplantation)	LA (600 mg) to the donor portal vein before ischemia and another LA (600 mg) at 15 min before reperfusion	Protecting against oxidative stress and reducing the appearance of postreperfusion syndrome	[74]
Human	Hepatic I/R (45 min /60 min) in resection	LA (600 mg, i.v) at 15 min before transection	Reducing I/R injury via attenuating apoptosis	[75]
Wistar albino rats	Hepatic I/R (45 min/60 min)	LA (100 mg/kg, i.p.) before ischemia and immediately before reperfusion period	Alleviating the I/R-induced liver injury in the hepatic structure and function via antioxidant properties	[76]
Wistar rats	Hepatic I/R (60 min/(1, 3, 6, and 12 h)	Injection with LA (25 mg) into the caudal vein at 15 min before reperfusion and 60 min after ischemia	Scavenging oxygen and nitrogen free radicals, increasing GSH levels, reducing inflammatory reaction mediated by NF-*κ*B P65 and MIP-2	[77]
Sprague-Dawley rats	Hepatic I/R (60 min/45,90 min)	LA (10 or 50 *μ*M) at 20 min before ischemia	Attenuating I/R injury via activating the PI3-kinase/Akt pathway	[78]
Brown Norway rats	Hepatic selective lobe I/R (10 min/10 min), then sustained I/R (90 min/4 h)	LA (500 *μ*mol, i.v) at 15 min before ischemia	Improving tolerance to ischemia via downregulating proapoptotic gene	[79]
Brown Norway rats	Hepatic I/R (90 min/1 h)	LA (120 mg) via the inferior vena cava at 15 min in advance	Attenuation of I/R by reducing necrosis and apoptosis	[80]
Brown Norway rats	Hepatic I/R (90 min/1 and 4 h)	LA (1.5 ml, 5,000 mol, i.v) at 15 min prior to ischemia	Attenuating I/R via reducing cell deaths from necrosis and apoptosis	[81]

**Table 4:** The effects of LA on cerebral I/R injury.

Subjects	I/R	Treatments	Effects and mechanisms	Ref
Sprague-Dawley rats	Left external carotid artery permanently occluded and left internal carotid artery I/R (90 min/24 h)	LA (50, 70, and 100 mg/kg, i.h) at 2 h before I/R	Attenuating I/R injury via reducing oxidative stress and caspase-dependent apoptosis	[94]
Sprague-Dawley rats	Middle cerebral artery selective I/R (30 s/30 s) before sustained I/R (2 h/24 h)	LA (50 mg/kg i.p) for 30 min before I/R	Exhibiting neuroprotection by attenuating neuroinflammation via inhibiting the TLR4/MyD88/NF-*κ*B pathway	[95]
Sprague-Dawley rats	Left middle cerebral artery I/R (2 h/1, 3, 7, 14, 21, 28, 35, 42, 49, and 56 d)	LA injection (20 mg/kg) via the left external jugular vein immediately after I/R	Exhibiting neurorestorative effects via insulin receptor activation, anti-inflammatory and antioxidant actions	[96]
Sprague-Dawley rats	Ligating external carotid arteries and the internal carotid artery I/R (2 h/24 h)	LA (10, 20,40, and 80 mg/kg) via the left external jugular vein after reperfusion	Promoting functional recovery via attenuating oxidative damage mediated by the Nrf2/HO-1 pathway	[97]
Sprague-Dawley rats	Right middle cerebral artery I/R (30 min/5.5 h)	LA (0.05, 0.5, 5.0, or 50 mg/kg, i.v) at 30 min	LA as a neuroprotectant via increasing protein expression of superoxide dismutase 2	[98]
Sprague-Dawley rats	Permanent distal middle cerebral artery occlusion-bilateral common carotid artery I/R (30 min/48 h)	LA (40 mg/kg, i.p) at 30 min after ischemia	Protective effects by LA via reducing phosphate mTOR	[99]
Albino rats	Bilateral femoral arteries I/R (3 h/1d)	LA (100 mg/kg, i.p) for 7 d after I/R	Ameliorating neuronal damages, maintaining the normal structure, surface area, and the number of light neurons in the pyramidal layer	[100]
Mouse primary brain endothelial cell and bEnd.3 cell	Oxygen glucose deprivation/reperfusion (6 h/4 h)	LA (1 mM) pre- and posttreatment for ischemia	Protecting against oxygen glucose deprivation/reperfusion injury via promoting the Akt/mTOR pathway	[101]
Sprague-Dawley rats	Bilateral carotid artery I/R (30 min/24 h)	LA (25 mg/kg, i.v) pretreatments	Improving survival and protecting the rat brain	[102]
Gerbils	Forebrain I/R (5 min/5 d)	LA (20 mg/kg∗d, i.p) for 7 d	Improving locomotor abilities and damages of the CA1 hippocampal pyramidal cells via antioxidant actions	[103]
C57blk mice	Internal carotid artery I/R (45 min/24 h)	LA (100 mg/kg, i.h)at 1.5 h before ischemia	Reducing stroke infarct volume via antioxidant actions	[104]

**Table 5:** The effects of LA on spinal I/R injury.

Species	I/R models	Treatments	Effects and mechanisms	Ref
New Zealand rabbits	Abdominal aorta I/R (30 min/48 h)	LA (100 mg/kg, i.p) at 5 min before I/R	Decreasing spinal I/R injury via reducing plasma and spinal levels of nitric oxide, glutathione, malondialdehyde, and advanced oxidation protein products	[105]
New Zealand rabbits	Abdominal aorta I/R (30 min/48 h)	LA (100 mg/kg, i.p) at 20 min before ischemia	Decreasing neuronal degeneration, axonal damage, and microglial and astrocytic infiltration via oxidative stress	[106]
Wistar rats	Abdominal aorta I/R (30 min/3 d)	LA (100 mg/kg, i.h) at 3 days before ischemia	Reducing neurologic injury by maintaining the oxidant/antioxidant balance	[107]
New Zealand rabbits	Abdominal aorta I/R (20 min/2 h)	LA (100 mg/kg) at 10 min before ischemia	Exhibiting antioxidant efficacy with no effect on oxidative stress	[108]

## Abbreviations

Akt: Protein kinase B
ALDH2: Aldehyde dehydrogenase 2
CNTF: Ciliary neurotrophic factor
DHLA: Dihydrolipoic acid
Erk1/2: Extracellular-regulated kinase 1/2
ET: Endothelin
GDNF: Glial-derived neurotrophic factor
GFAP: Reducing levels of glial fibrillary acidic protein
HO-1: Heme oxygenase-1
LA: Lipoic acid
MIP-2: Macrophage inflammatory protein-2
mTOR: Mammalian target of rapamycin
MyD88: Myeloid differentiation primary response 88
Nrf2: Nuclear factor erythroid 2-related factor 2
p38 MAPK: Mitogen-activated protein kinase p38
PI3K: Phosphatidylinositol 3-kinase
PKC: Protein kinase C
TLR4: Toll-like receptor 4.
